# The complete chloroplast genome and phylogenetic analysis of *Rubus sumatranus* Miq 1861 (Roseaceae)

**DOI:** 10.1080/23802359.2024.2438277

**Published:** 2024-12-07

**Authors:** Wei Guo, Longyuan Wang, Wei Wu

**Affiliations:** College of Horticulture and Landscape Architecture, Zhongkai University of Agriculture and Engineering, Guangzhou, Guangdong, China

**Keywords:** Chloroplast genome, next-generation sequencing, phylogenomics, Rosaceae, wild raspberry

## Abstract

The wild raspberry species *Rubus sumatranus* Miq 1861 is a promising resource for breeding thermotolerant cultivars. Its complete chloroplast genome spans 155,935 base pairs (bp), featuring the classic quadripartite structure: an 18,729 bp small single-copy region, an 85,662 bp large single-copy region, and two 25,772 bp inverted repeats. A total of 130 genes were identified, including 86 protein-coding, 36 tRNA genes, and 8 rRNA genes. A maximum likelihood phylogenetic tree based on chloroplast genomes shows that *R. sumatranus*, within the subgenus *Idaeobatus*, is sister to the subgenus *Batothamnus*. This confirms the polyphyletic nature of the subgenus *Idaeobatus*. The chloroplast genome assembly of *R. sumatranus* enhances our understanding of its evolutionary history.

## Introduction

The *Rubus* L. genus, within the Rosoideae subfamily of the Rosaceae, is cosmopolitan, with a notable prevalence in temperate zones. Comprising around 740 species, the genus was traditionally divided into twelve subgenera (Focke 1910, 1911, 1914). However, recent morphological and molecular evidence suggests a reduction to ten subgenera (Huang et al. 2023). The subgenera *Idaeobatus*, *Rubus*, and *Malachobatus* contain the most species. *Rubus* is one of the most genetically complex genera due to interspecific hybridization, polyploidy, and apomixis (Thompson 1997; Alice et al. 2001). Significant morphological variation within species presents challenges for classification (Robertson 1974). Recent studies, restricted by inadequate sampling and a narrow selection of molecular markers, have left the phylogeny at subgeneric and sectional levels unresolved (Wang et al. 2016; Carter et al. 2019; Wang et al. 2019; Huang et al. 2023).

**Figure 1.**
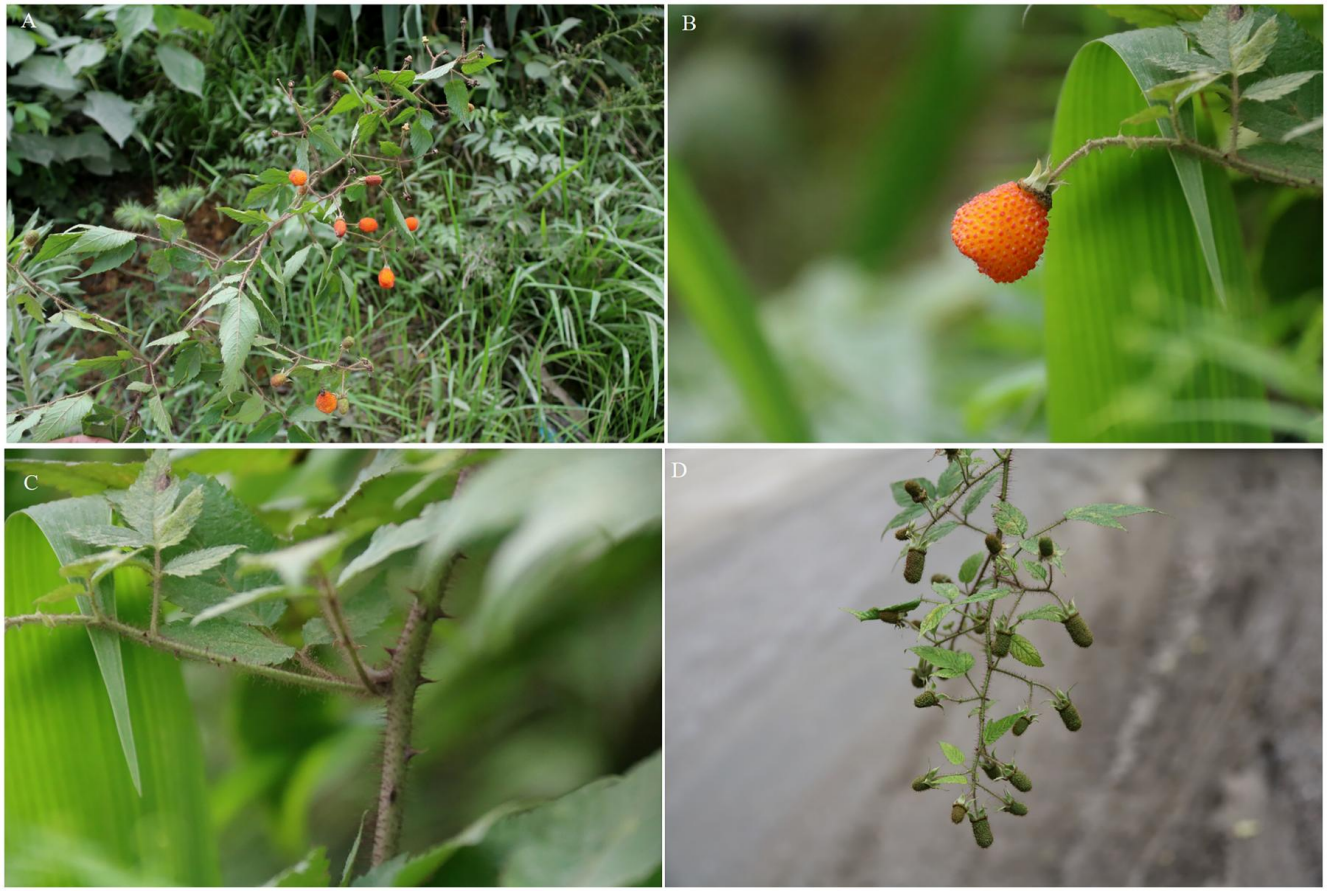
*Rubus sumatranus* growing in its natural habitat. A – the whole plant; B – aggregate fruit; C – stems with purplish-red glandular hairs, soft hairs, and bristles; D – infructescence. The photo was taken and redacted by GW & WW at Mount Yuanbao in Rongshui County, Guangxi Province (25°22′36.8″N, 109°08′23.2″E).

*Rubus sumatranus*, a shrub that grows erect or scrambles up to 2 meters high, is native to the warm temperate and subtropical regions of East Asia, including southern China, Japan, and extending to northeast India, Thailand, Laos, Vietnam, Malaysia, and Indonesia. Historically, it was considered synonymous with *Rubus pirifolius* Smith (Focke 1910). The plant features branches, rachises, petioles, pedicels, and inflorescences covered with purplish-red glandular hairs, soft hairs, and bristles (Lu and Boufford 2003) (Figure 1). Classified under the subgenus *Idaeobatus*, *R. sumatranus* is notable for its abundant orange-red aggregate fruits. Its roots are used in traditional medicine for their heat-clearing, detoxifying and diuretic properties (Lu and Boufford 2003). Given its broad distribution, *R. sumatranus* is a valuable resource for adaptability breeding programs, particularly for developing thermotolerant cultivars. Its resilience across various climates makes it an excellent candidate for genetic studies aimed at enhancing the productivity of cultivated varieties. So far, no genetic resources are available for this species. As a first step toward utilizing this species, we characterized the chloroplast genome and determined the phylogeny placement for *R. sumatranus*.

## Materials and methods

### Plant material collection and DNA extraction

Fresh leaves from an individual of *R. sumatranus* were collected at Yuanbao Mountain National Natural Reserve in Rongshui County, Guangxi Province (25°22′36.8″N, 109°08′23.2″E). The voucher specimen is deposited at the SYSU Herbarium (Contact person: Wei Wu; e-mail: wuei06@zhku.edu.cn) under accession number GXYBS0021. Total genomic DNA was extracted using a modified cetyltrimethyl ammonium bromide (CTAB) method (Doyle and Doyle 1987) and quantified with a Qubit^™^ 4.0 Fluorometer (Fisher Scientific, USA).

### Library construction and sequencing

A DNA library with 350–550 bp insert size library was constructed using 1.0 μg DNA according to the Illumina’s protocol and sequenced on an Illumina Novaseq 6000 platform (Illumina, USA) using paired-end 150 bp reads.

### Sequence assembly and annotation

After trimming adaptors, raw Illumina reads were filtered using fastp v0.23.4 with default settings (Chen et al. 2018). Pair-end clean reads of *R. sumatranus* were processed through the GetOrganelle v1.7.7.0 pipeline for plastome assembly using the parameter ‘-R 30 -k 65,85,105,115 -F embplant_pt’ (Jin et al. 2020). Chloroplast genes were annotated with two reliable software tools: CpGAVAS (Liu et al. 2012) and GeSeq v2.03 (Tillich et al. 2017), simultaneously. The annotated chloroplast genome was manually verified and corrected by comparison with the published plastome of *R. leucanthus* (GenBank accession number: MK105853.1; Guo et al. 2019). The chloroplast structure and gene order were visualized using CPGView (Liu et al. 2023).

### Phylogenetic tree construction

To determine the phylogenetic position of *R. sumatranus*, we selected complete plastome sequences from 11 other *Rubus* species across six subgenera. Before reconstructing the phylogenetic tree, 82 shared unique CDS sequences from each species were extracted using BEDTools (Quinlan 2014), aligned with MAFFT v7.487 (Katoh and Standley 2013), and concatenated using AMAS (Borowiec 2016). A maximum likelihood (ML) phylogenetic tree was constructed based on the best-fit model of TVM+F + R5, determined by BIC, with IQ-TREE v2.0 (Nguyen et al. 2015) and 1,000 bootstrap replicates. Two closely related species, *Fragaria vesca* subsp*. vesca* (Rosaceae; GenBank accession number: NC_015206.1; Shulaev et al. 2011) and *Geum aleppicum* (Rosaceae; GenBank accession number: NC_060733.1; Zhang et al. 2022), were included as outgroups.

## Results

Out of 10.0 GB of raw Illumina pair-end reads, 9.85 GB of clear reads were obtained for the *R. sumatranus* chloroplast genome assembly. The raw reads were mapped to the final assembly, revealing substantial sequencing coverage. The base depth ranged from 1566 × to 6578×, with a mean base depth of 4567×, ensuring high accuracy (see Supplementary material, Figure S1).

**Figure 2.**
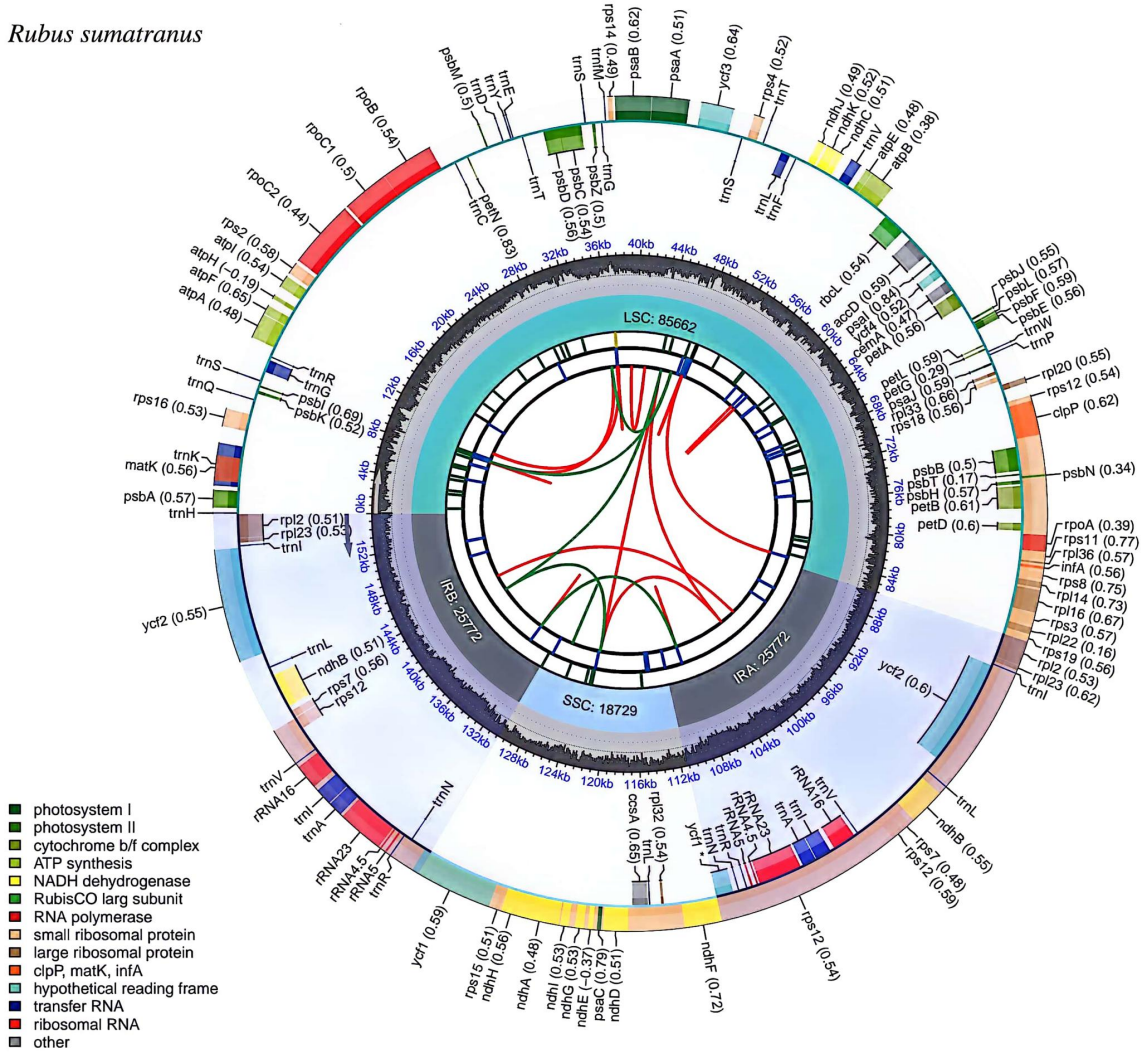
Circular chloroplast genome map of *Rubus sumatranus*. The innermost track displays dispersed repeats, including direct (red) and palindromic (green) repeats. The next track shows long tandem repeats as short blue bars. The third track depicts short tandem repeats/microsatellites using colored bars, where the color indicates the repeat unit size (black: complex, green: 1 bp, yellow: 2 bp, purple: 3 bp, blue: 4 bp, orange: 5 bp, red: 6 bp). The fourth track outlines the small single-copy (SSC), inverted repeat (IRa and IRb), and large single-copy (LSC) regions. The fifth track plots GC content, and the base frequency is shown between the fourth and fifth tracks. The outermost track displays the genes, color-coded by functional category, with transcription direction indicated (clockwise/counterclockwise). The functional classifications are provided in the bottom left corner.

The *R. sumatranus* chloroplast genome, spanning 155,935 bp, exhibits the typical quadripartite structure found in other seed plants (Jansen and Ruhlman 2012). It includes two 25,772-bp inverted repeat regions, an 85,662-bp large single-copy region, and an 18,729-bp small single copy region (Figure 2). A total of 130 genes were predicted: 86 protein-coding, 36 tRNA, and 8 rRNA genes. Among these, 18 genes contain two exons and 4 genes contain three exons. The *rps*12 genes undergo trans-splicing, as confirmed by CPGView (https://www.1kmpgcn/cpgview/) (see Supplementary Figures S2 and S3). The overall GC content is 37.03%, with 31.08% A, 18.87% C, 31.89% T and 18.16% G. The plastome sequence is deposited in the NCBI GenBank database under accession number PP737792.1.

**Figure 3.**
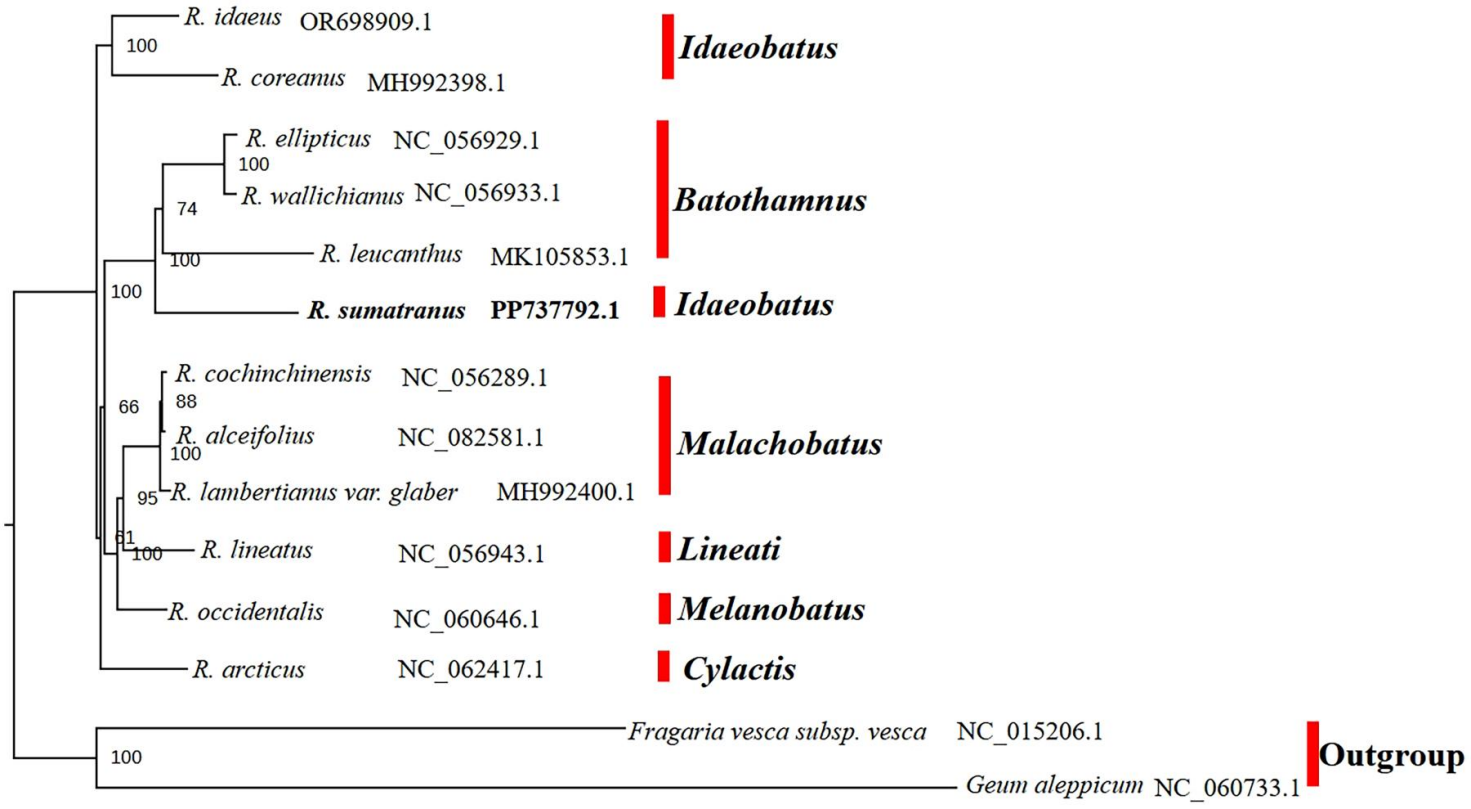
The maximum likelihood tree of 12 *Rubus* species and two outgroups based on chloroplast genome sequences. The 12 species were *R. idaeus* (OR698909.1), *R. coreanus* (MH992398.1) (Chen et al. 2019), *R. ellipticus* (NC_056929.1) (unpublished), *R. wallichianus* (NC_056933.1) (unpublished), *R. leucanthus* (MK105853.1) (Guo et al. 2019), *R. sumatranus* (PP737792.1), *R. cochinchinensis* (NC_056289.1) (Chen et al. 2020), *R. alceifolius* (NC_082581.1) (unpublished), *R. lambertianus* var. *glaber* (MH992400.1) (unpublished), *R. lineatus* (NC_056943.1) (unpublished), *R. occidentalis* (NC_060646.1) (unpublished), *R. arcticus* (NC_062417.1), two outgroups *Fragaria vesca* subsp. *Vesca* (NC_015206.1) (Shulaev et al. 2011), *Geum aleppicum* (NC_060733.1) (Zhang et al. 2022). Bootstrap supports were calculated from 1000 replicates. The *R. sumatranus* was labeled in bold font in the phylogenetic tree, and the subgenus position of *Rubus* species were marked with red bar next to it.

Maximum likelihood phylogenetic analysis revealed that *R. sumatranus* is sister to the subg*. Batothamnus* (*R. leucanthus*, *R. ellipticus*, *R. wallichianus*) with strong support, and subg*. Idaeobatus* is not monophyletic (Figure 3).

## Discussion and conclusion

Due to extensive morphological variations, *R. sumatranus* has been treated as synonyms with *Rubus croceacanthus* **H. Lév** 1912, *Rubus glandulosocalycinus* Hayata 1915, *Rubus dolichocephalus* Hayata 1913, respectively (Huang and Hu 2009). The phylogenetic position of *R. sumatranus* remains unresolved. For example, it was placed in the sect. *Rosifolii* in subg. *Idaeobatus (*Kikuchi et al. 2022). A recent study, the phylogenetic position of *R sumatranus* was inconsistent between chloroplast genome and nuclear genomes, suggesting incomplete lineage sorting or hybridizations associated with *R. sumatranus* (Gao et al. 2023). Our preliminary study shows *R. sumatranus* is sister to the subg*. Batothamnus*, with subg. *Idaeobatus* being non-monophyletic, confirming previous findings (Wang et al. 2016; Carter et al. 2019). Given its wide distribution and polymorphic traits, more extensive sampling and informative markers are needed to clarify its taxonomy and phylogenetic position.

Overall, this study characterized the chloroplast genome of *R. sumatranus* and determined its phylogenetic position, providing valuable genomic resources for future breeding efforts involving this wild raspberry species.

## Supplementary Material

Fig_S2_600dpi.jpg

Fig_S3_600dpi.jpg

Fig_S1_600dpi.jpg

## Data Availability

The chloroplast genome sequence data in this study are openly available in GenBank of NCBI at https://www.ncbi.nlm.nih.gov/ under the accession number PP737792. Raw sequencing reads used here have been deposited in the SRA database of NCBI under accession number SRR28810725. The associated ‘BioProject’, and ‘Bio-Sample’ numbers are PRJNA1104367 and SAMN41075590, respectively.
