# Supplementary figures and images for: The complete chloroplast genome and phylogenetic analysis of Rubus sumatranus Miq 1861 (Roseaceae)
Source: Mitochondrial DNA B Resour. 2024 Dec 7;9(12):1669–73. doi: 10.1080/23802359.2024.2438277 (PMC11626868; doi:10.1080/23802359.2024.2438277)

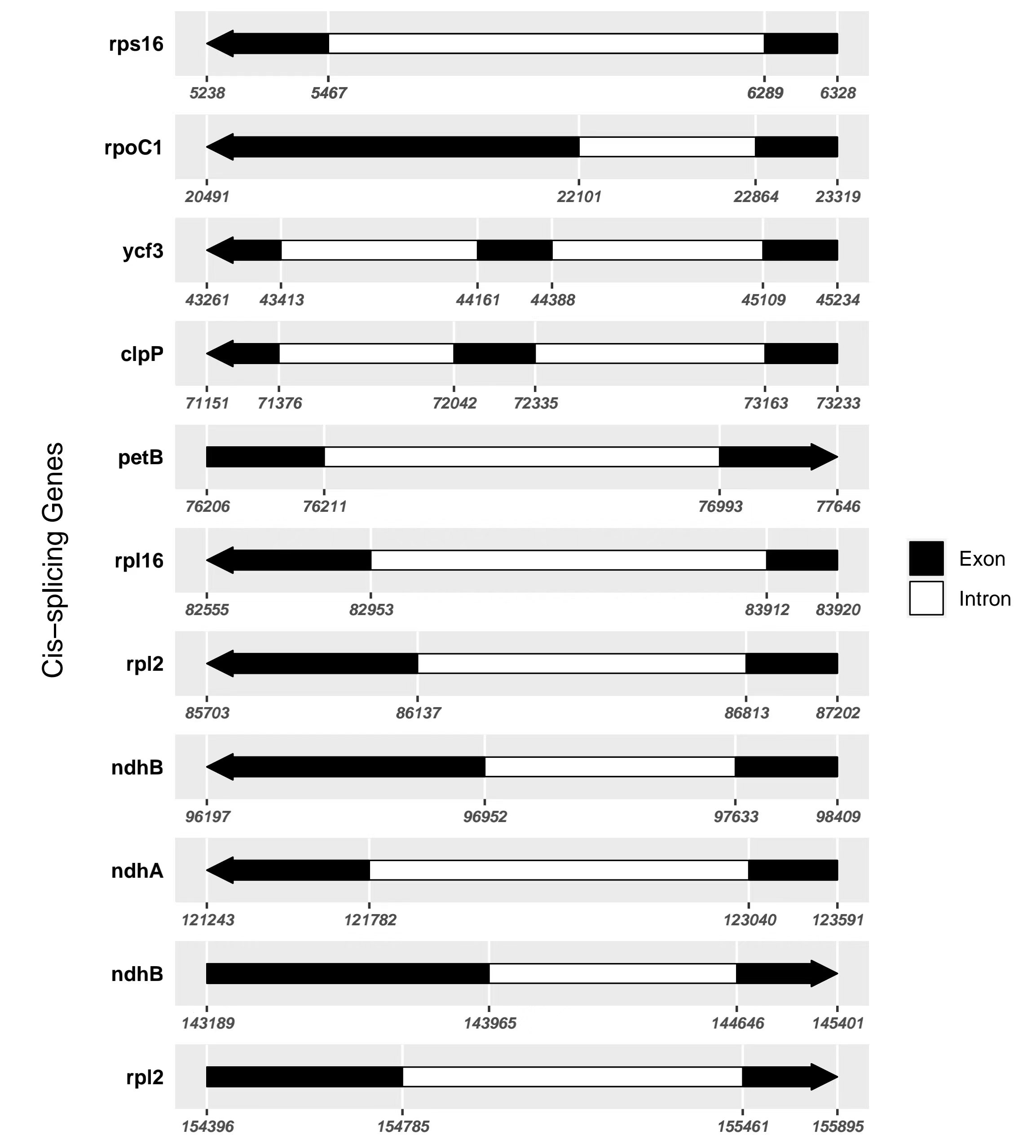

Supplement: Fig_S2_600dpi.jpg [file TMDN_A_2438277_SM0234.jpg]

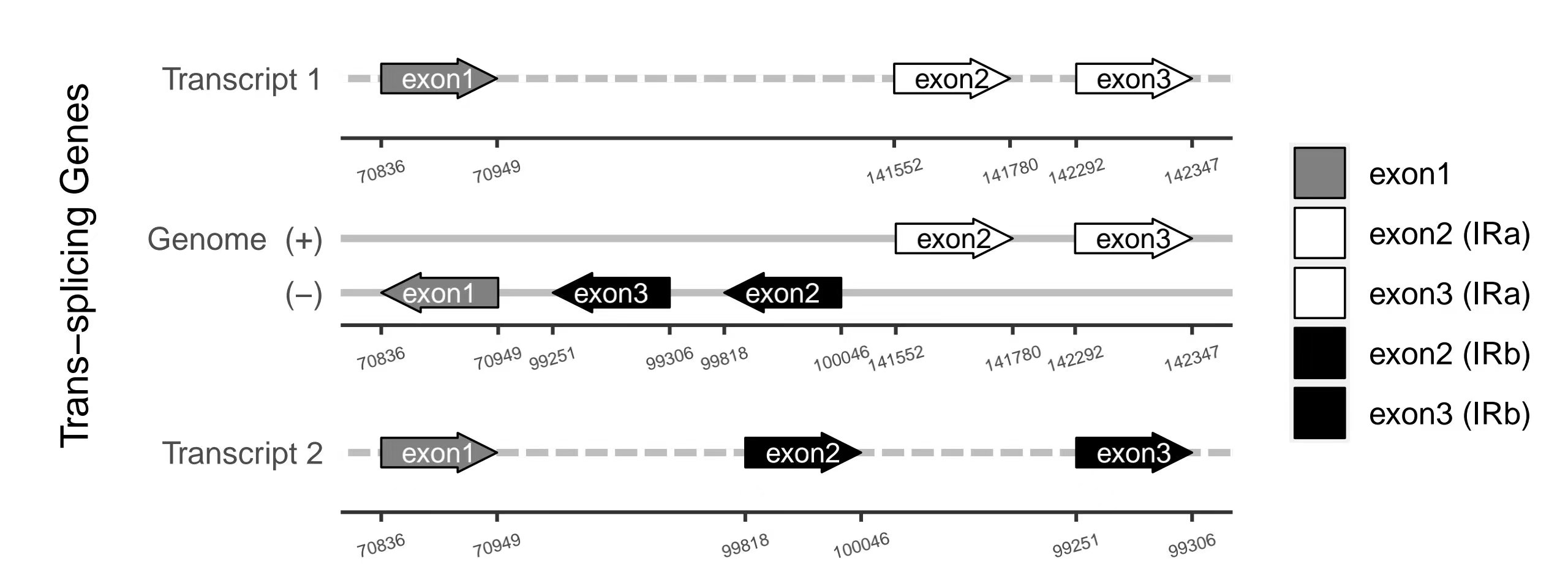

Supplement: Fig_S3_600dpi.jpg [file TMDN_A_2438277_SM0233.jpg]

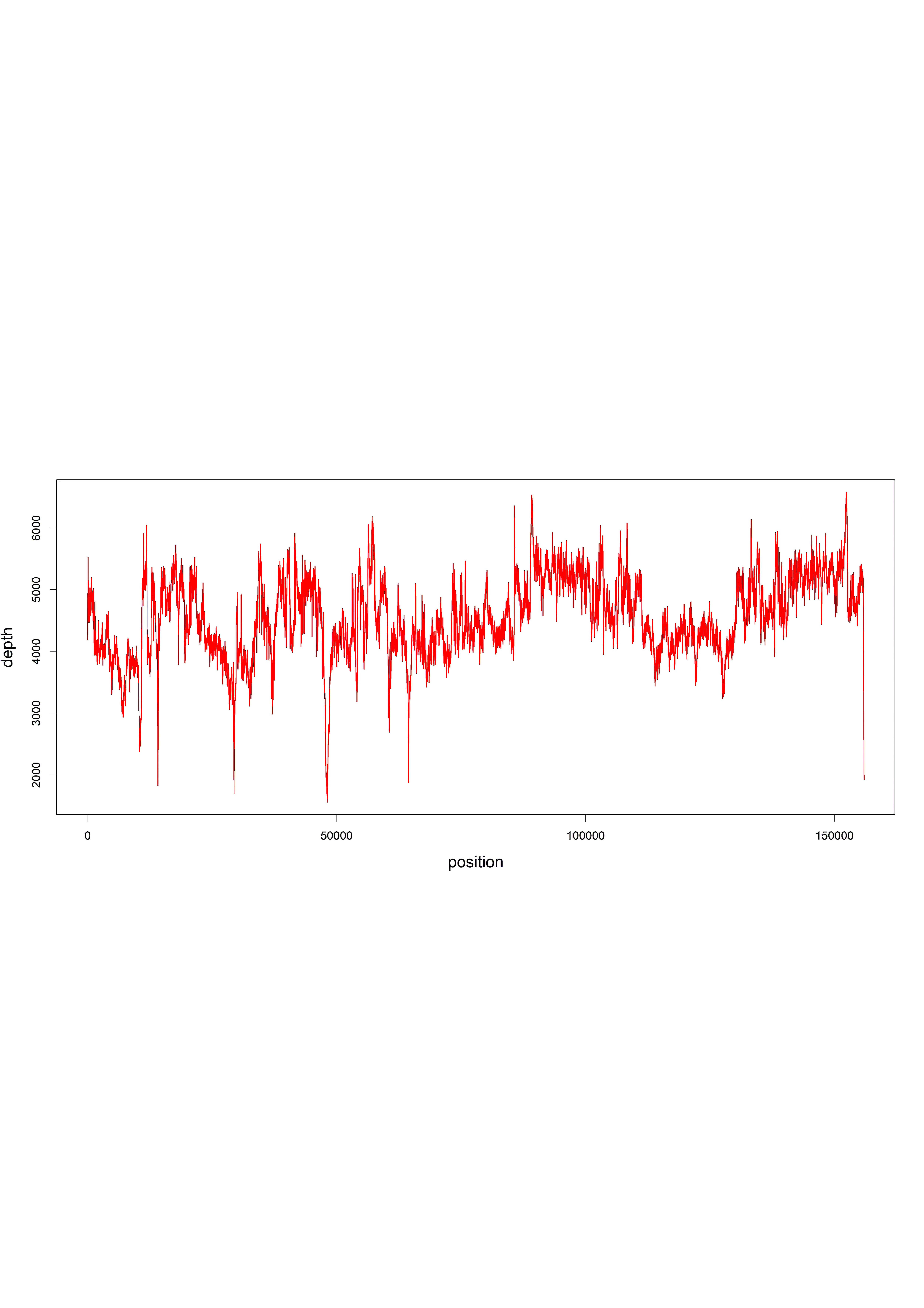

Supplement: Fig_S1_600dpi.jpg [file TMDN_A_2438277_SM0232.jpg]
